# Molecular Epidemiology of HIV-1 in Jilin Province, Northeastern China: Emergence of a New CRF07_BC Transmission Cluster and Intersubtype Recombinants

**DOI:** 10.1371/journal.pone.0110738

**Published:** 2014-10-30

**Authors:** Xingguang Li, Xihui Zang, Chuanyi Ning, Yi Feng, Cunxin Xie, Xiang He, Yutaka Takebe, Liuyan Sun, Qi Guo, Hui Xing, Marcia L. Kalish, Yiming Shao

**Affiliations:** 1 State Key Laboratory for Infectious Disease Prevention and Control, National Center for AIDS/STD Control and Prevention, Chinese Center for Disease Control and Prevention, Beijing, China, and Collaborative Innovation Center for Diagnosis and Treatment of Infectious Diseases, Hangzhou, Zhejiang, China; 2 HIV Molecular Epidemiology and Virology Research Group, The State Key Laboratory of Virology, Wuhan Institute of Virology, University of Chinese Academy of Sciences, Wuhan, Hubei, China; 3 Jilin Provincial Center for Disease Control and Prevention, Changchun, Jilin, China; 4 AIDS Research Center, National Institute of Infectious Diseases, Tokyo, Japan; 5 Vanderbilt Institute for Global Health, Vanderbilt University School of Medicine, Nashville, Tennessee, United States of America; 6 Guangxi Key Laboratory of AIDS Prevention and Treatment & School of Public Health, Guangxi Medical University, Nanning, Guangxi, China; Institut Pasteur of Shanghai, Chinese Academy of Sciences, China

## Abstract

**Objective:**

To investigate the HIV-1 molecular epidemiology among newly diagnosed HIV-1 infected persons living in the Jilin province of northeastern China.

**Methods:**

Plasma samples from 189 newly diagnosed HIV-1 infected patients were collected between June 2010 and August 2011 from all nine cities of Jilin province. HIV-1 nucleotide sequences of *gag* P17–P24 and *env* C2–C4 gene regions were amplified using a multiplex RT-PCR method and sequenced. Phylogenetic and recombination analyses were used to determine the HIV-1 genotypes.

**Results:**

Based on all sequences generated, the subtype/CFR distribution was as follows: CRF01_AE (58.1%), CRF07_BC (13.2%), subtype B’ (13.2%), recombinant viruses (8.1%), subtype B (3.7%), CRF02_AG (2.9%), subtype C (0.7%). In addition to finding CRF01_AE strains from previously reported transmission clusters 1, 4 and 5, a new transmission cluster was described within the CRF07_BC radiation. Among 11 different recombinants identified, 10 contained portions of gene regions from the CRF01_AE lineage. CRF02_AG was found to form a transmission cluster of 4 in local Jilin residents.

**Conclusions:**

Our study presents a molecular epidemiologic investigation describing the complex structure of HIV-1 strains co-circulating in Jilin province. The results highlight the critical importance of continuous monitoring of HIV-infections, along with detailed socio-demographic data, in order to design appropriate prevention measures to limit the spread of new HIV infections.

## Introduction

In China, it is estimated that 780,000 people were living with HIV by the end of 2011, according to the “China AIDS Response Progress Report” [1]. China is experiencing a dynamic and complex HIV/AIDS epidemic. The reported predominant co-circulating HIV-1 genotypes are: subtype B’, circulating recombinant form (CRF) CRF01_AE, CRF07_BC, and CRF08_BC [2], [3]. These three CRFs and the B’ subtype constituted 92.8% of reported HIV-1 infections in China in 2006 based on our nationwide molecular epidemiology survey, and were detected in all high-risk groups including former plasma donors (FPDs), injecting drug users (IDUs), men having sex with men (MSM) and heterosexual transmissions [2]. Co-circulation with strains from different HIV-1 subtypes, CRFs, and unique recombinant forms (URFs) in these risk groups can create opportunities for the emergence of new, hybrid recombinants [4]–[7].

Jilin province is located in the center of northeast China, bordering with Russia to the east, the Democratic People’s Republic of Korea (DPRK, also referred to as North Korea) across the rivers of Yalu and Tumen to the southeast, Liaoning province to the southwest, Inner Mongolia Autonomous Region to the west, and Heilongjiang province to the north. Jilin province encompasses an area of 187,400 square kilometers, and is divided into nine regions: Changchun (the capital of Jilin province), Jilin (located in the center of Jilin province), Siping, Tonghua, Baishan, Liaoyuan, Baicheng, Songyuan and Yanbian Korean Autonomous Prefecture. According to the sixth nationwide population census of 2010 [8], Jilin province had a population of 27,462,297, with a total of 44 ethnicities, including Han, Manchu, Mongol and Hui. Jilin’s central location contributes to a persistent influx and outflow of people, mainly due to trade, labor, tourism, and education; this increasingly mobile population provides an increased opportunity for importing new HIV-1 strains and increasing transmissions.

The first known AIDS case in Jilin province was in a laborer infected through heterosexual contact with a female commercial sex worker (CSW) in Mombasa, Kenya in 1993, however, the HIV genotype of the infected patient was unknown. Jilin province experienced a low level of new HIV infections between 1993 and 1994, and then the number of reported HIV infections increased yearly [8]. A total of 1,477 HIV infections had been reported by the end of 2010 among all risk groups [9].

Between 2004 and 2010, the major risk for HIV-1 infections changed from FPDs (2.09% in 2010 compared with 42.19% in 2004) to those infected through sexual transmission (93.4% in 2010 compared with 35.9% in 2004) [10]. In addition, the proportion of HIV-1 infections among MSM surged approximately 28-fold: from 1.8% (2/114) in 2005 to 48.6% (139/286) in 2010 [10]. However, the most heavily affected regions in Jilin province were still concentrated in several big regions, including Changchun, Jilin and Yanbian Korean Autonomous Prefecture [9], [10].

The current distribution of HIV-1 subtypes, CRFs, and recombinants in Jilin province is largely unknown. Therefore, a detailed HIV-1 molecular epidemiologic investigation to determine the genotypic distribution and the emergence and spread of new subtypes and CRFs is of great importance for understanding the dynamics of the HIV-1 epidemic in this region. In the present study, we performed an HIV-1 molecular epidemiological investigation of 189 newly diagnosed HIV-infections in Jilin province.

## Methods

### Study subjects and dataset information

A total of 189 newly diagnosed HIV-infected persons identified between January 2008 and December 2010 at local voluntary counseling and testing sites (VCT), sentinel surveillance sites, and medical institutions in Jilin province were agreed to be enrolled in this study. All newly diagnosed HIV-infected people were identified from various cities and risk groups. Whole blood samples were collected in 2010 (n = 93) and 2011 (n = 96); plasma was separated and stored at –80°C. The study was approved by the institutional review board of the National Center for AIDS/STD Control and Prevention, China CDC. A written informed consent, as well as a socio-demographic questionnaire, was obtained from each participant in this study.

The socio-demographic data that was collected included sex, age, ethnicity, marital status, education background, year of diagnosis, year of sampling, site of sampling, CD4+ T Cell Count and risk group.

### HIV-1 RNA extraction, amplification and sequencing

Plasma samples from 189 newly diagnosed HIV infected participants were collected and submitted for RT-PCR and sequencing. Viral RNA was extracted from 280 µl of plasma using the QIAamp Viral RNA Mini kit (Qiagen, Valencia, California, USA) following the manufacturer’s instructions [11], [12]. The extracted viral RNA was subjected to a multiplex reverse transcription, polymerase chain reaction (RT-PCR) to obtain the nucleotide sequences of HIV-1 *gag* P17–P24 (HXB2: position 781-1836 for 1056 base pairs (bp)) and *env* C2–C4 (HXB2: positions 7002-754 for 540 bp) gene regions as previously described [13].

The positive PCR products were purified using QIAquick Gel Extraction Kit (Qiagen, Valencia, California, USA) and sequenced directly on an ABI 3730XL automated sequencer using BigDye terminators (Applied Biosystems, Foster City, California, USA) by Beijing Biomed Technology Development CO., Ltd (Beijing, China).

### Phylogenetic tree and recombination analysis

The nucleotide sequences of the 134 HIV-1 *gag* P17–P24 and 121 *env* C2–C4 gene regions were aligned separately using the Gene Cutter software (http://www.hiv.lanl.gov/cotent/sequence/.html) with HIV-1 group M subtype reference sequences [Los Alamos National Laboratory (LANL) HIV Sequence Database (http://www.hiv.lanl.gov/cotent/sequence/NEWALIGN/align.html, accessed in April 2013)] as follows: A1 (3), A2 (3), B (4), C (4), D (4), F1 (4), F2 (4), G (4), H (4), J (3), K (2), CRF01_AE (2), CRF02_AG (3), CRF08_BC (1) and group N (3). In addition to these 48 reference strains, we used 62 reference sequences from viruses that represent subtypes/CRFs commonly identified in China as follows: subtypes A1 (1), B (6), B’ (7), C (2), CRF07_BC (5) and CRF01_AE (39), CRF08_BC (2). Both *gag* and *env* sequences from these 110 reference strains were available. Following alignment, manual adjustments were made taking into consideration protein coding sequences using BioEdit software [14], [15]. Neighbor-joining phylogenetic trees were constructed using the Kimura 2-parameter model of evolution, including both transitions and transversions [16], implemented in the MEGA 5.0 software package [17]. The reliability of the tree structure or branching order was evaluated by bootstrap analysis with 1000 replicates [18]. In order to better display the phylogenetic trees of the HIV-1 *gag* P17–P24 and *env* C2–C4 regions, the sequences were separated into two different Neighbor-joining trees, one containing only the subtype A, CRF01_AE, CRF02_AG-related sequences and the other representing the B/B’, C, CRF07_BC related sequences. If there was evidence of recombination in any of the sequences (i.e., discordant gene regions or outlier position in a tree), they were further analyzed using the jumping profile Hidden Markov Model program (jpHMM; http://jphmm.gobics.de/) [19]. In order to confirm the possible recombinant structures and identify recombinant breakpoints of the potential HIV-1 recombinants, Bootscanning analysis was performed using Simplot 3.5.1 software package with window size of 300 bp, step size of 20 bp) [20].

### Nucleotide sequence accession numbers

All the nucleotide sequences obtained in this study were submitted to GenBank under accession numbers of KF818784–KF818917 for the HIV-1 *gag* P17–P24 gene region and KF818661–KF818713, KF818715–KF818772, KF818774–KF818783 for the HIV-1 *env* C2–C4 gene region.

## Results

### Demographic and epidemiologic information on the study participants

A total of 189 newly diagnosed HIV-1 infected samples were collected from local voluntary counseling and testing sites (VCT), sentinel surveillance sites and medical institutions in Jilin province and were used for the HIV-1 genetic analysis. For each sample, *gag* P17–P24 and *env* C2–C4 genes were amplified and sequenced. From the 189 plasma samples, 134 *gag* P17–P24 and 121 *env* C2–C4 gene sequences were obtained; 119 samples had both *gag* P17–P24 and *env* C2–C4 sequences; a total of 136 samples were genotyped with a success rate of 72.0% (136/189). The failure of PCR amplification or sequencing was likely related to factors such as poor transportation and storage conditions, low plasma volumes, low viral load, repeated freezing and thawing, and poor amplification and/or sequencing primer specificity.

The demographic and epidemiologic data are summarized in Table S1. Among the 189 participants, 81.5% were males. The mean age of the participants was 37.3±12.9 years. 88.4% (167/189) of participants were of Han nationality and 11.6% (22/189) of participants were minority nationalities, including Korean, Manchu, Dai, Yi, Hui, Lisu, and Mongol. The median CD4+ T Cell Count was 380 cells/µl, suggesting that most of these newly diagnosed participants had chronic infections. The following risk groups were identified: MSM (107/189, 56.6%); heterosexuals (65/189, 34.4%); FPDs (9/189, 94.8%); IDUs (3/189, 1.6%); blood transfusion recipients (2/189, 1.1%); mother-to-child transmission (1/189, 0.5%); unknown (2/189, 1.1%) and geographical regions in Jilin province: Changchun (125/189, 66.1%); Jilin (20/189, 10.6%); Yanbian Korean Autonomous Prefecture (15/189, 7.9%); Tonghua (10/189, 5.3%); Liaoyuan (4/189, 2.1%); Baishan (4/189, 2.1%); Baicheng (4/189, 2.1%); Songyuan (4/189, 2.1%); and Siping (3/189, 1.6%).

### HIV-1 genotype distribution of the study participants

**Figure 1:**
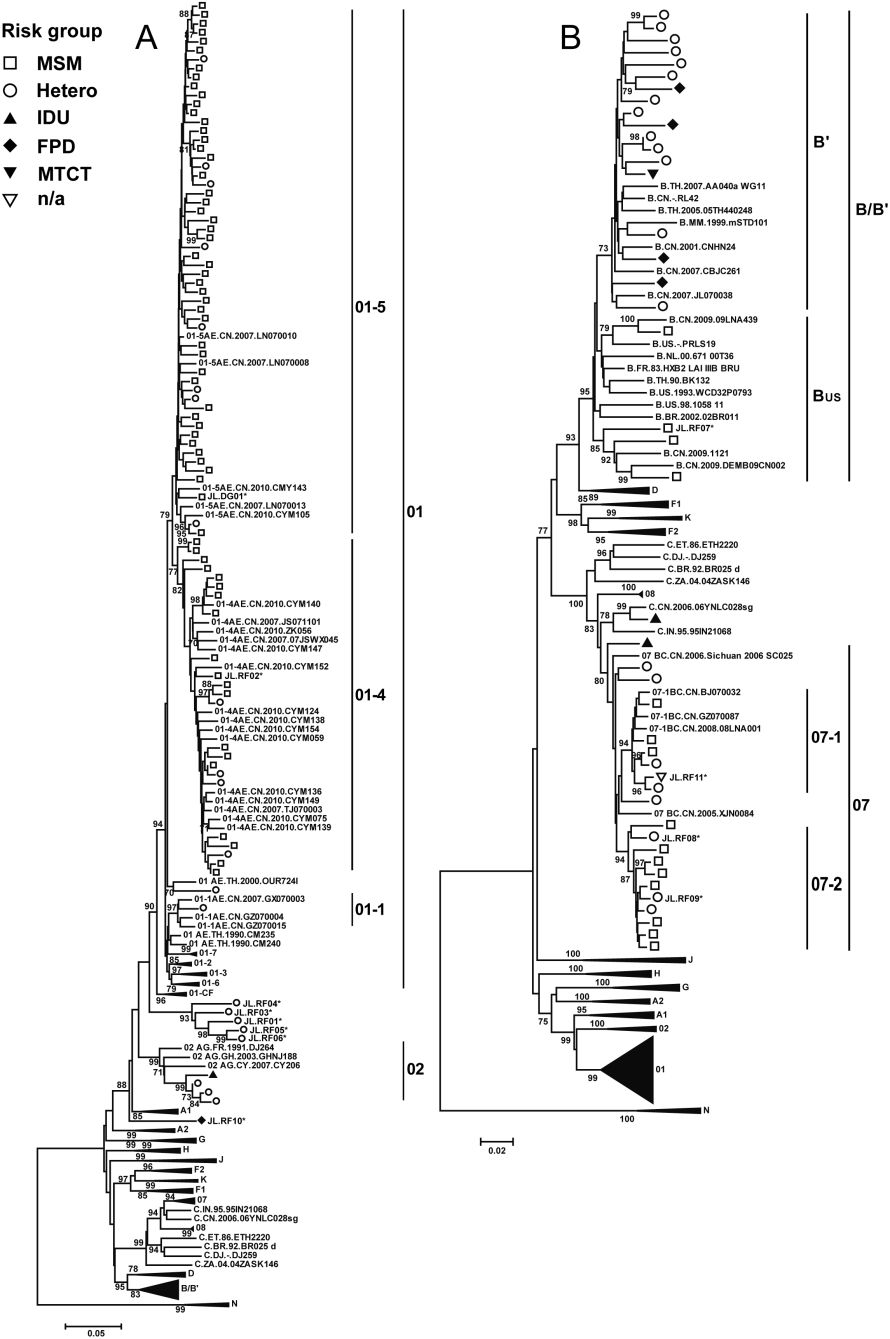
Neighbor-joining phylogenetic tree of HIV-1 *gag* P17–P24 gene of study samples from the Jilin province of northeastern China. Neighbor-joining phylogenetic trees were constructed for 134****DNA sequences of HIV-1 *gag* P17–P24 and reference sequences. The 134 *gag* P17–P24 gene sequences were separated into two different neighbor-joining trees, Fig. 1A containing only the subtype A, CRF01_AE, CRF02_AG-related sequences and Fig. 1B representing the B/B’, C, CRF07_BC related sequences. The stability of each node was assessed by bootstrap analyses with 1000 replicates and only bootstrap values ≥70 are shown at the corresponding nodes. CRF01_AE, CRF02_AG, CRF07_BC and CRF08_BC are labeled CRF01, CRF02, CRF07 and CRF08, respectively, for simplicity. The seven unique CRF01_AE lineages detected in China are labeled CRF01-1 through CRF01-7 and the Central African CRF01_AE sequences are labeled CRF01-CF. The two distinct CRF07_BC lineages identified among MSM are labeled CRRF07-1 and CRF07-2. The asterisk (*) next to sequence name indicates the identified recombinant or discordant genotype in the study. The symbols representing the risk groups are shown at the top-left corner of the figure.

**Figure 2:**
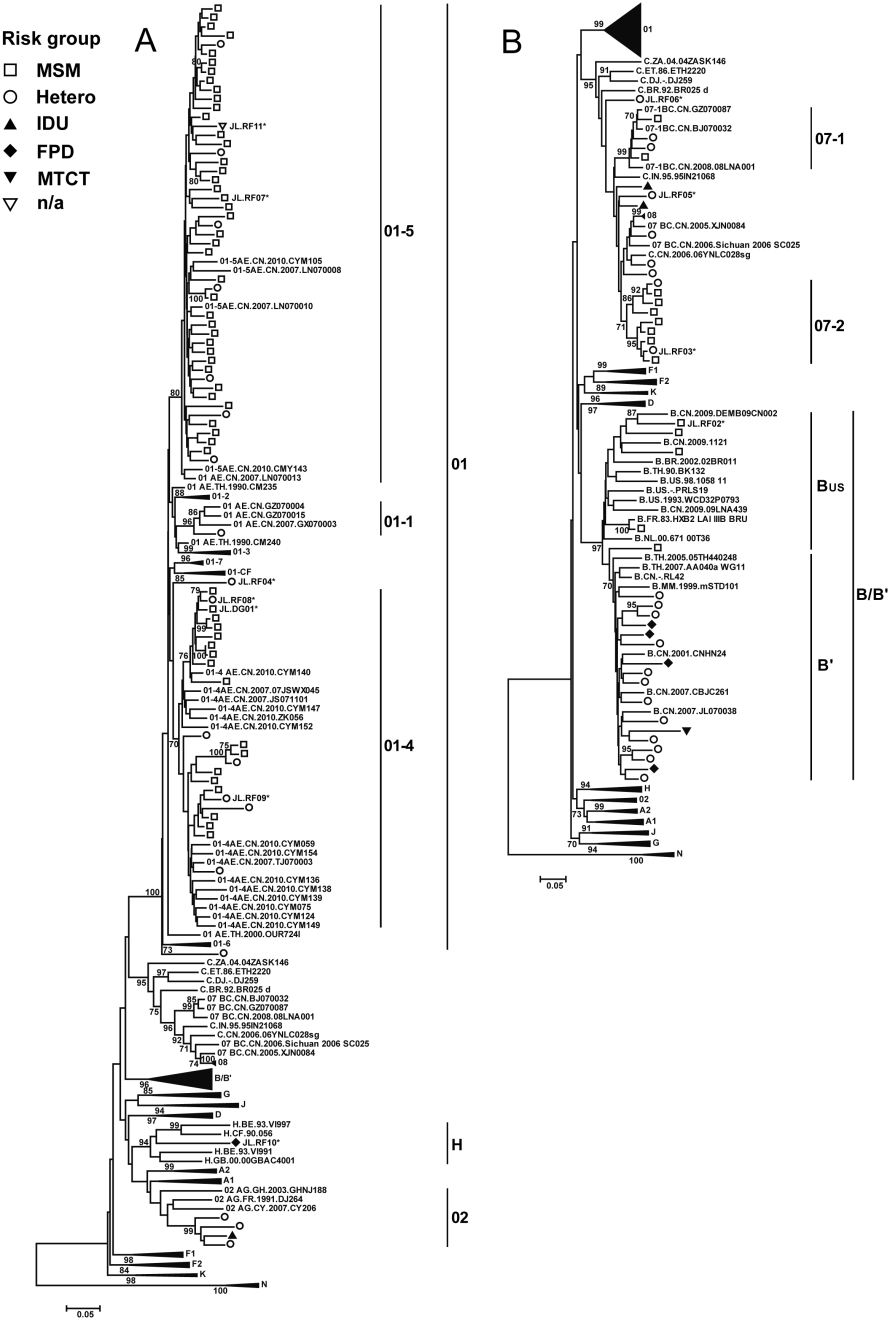
Neighbor-joining phylogenetic tree of HIV-1 *env* C2–C4 gene of study samples from the Jilin province of northeastern China. Neighbor-joining phylogenetic trees were constructed for 121 nucleotide sequences of HIV-1 *env* C2–C4 and reference sequences. The phylogenetic trees were constructed using methods described in Figure 1.

Phylogenetic analyses of the nucleotide sequences of 134 *gag* P17–P24 and 121 *env* C2–C4 gene regions are shown in Fig. 1 and Fig. 2, respectively. A total of 136 HIV-1 samples were successfully amplified and genotyped for at least one gene region, and most for both gene regions (Table 1 and Table S2). The classifications for the 119 samples having both *gag* and *env* sequences (*gag*/*env*) were as follows: CRF01/CRF01 (69/119, 58.0%), subtype B’/B’ (17/119, 14.3%), CRF07/CRF07 (16/119, 13.4%), CRF02/CRF02 (4/119, 3.4%), subtype B/B (2/119, 1.7%), and subtype C/C (1/119, 0.8%). Furthermore, there were 10 (8.4%) samples with discordant *gag*//*env* phylogenies, and 2 of these showed complex levels of recombination involving gene regions from at least 3 different subtypes/CRFs: CRF07//CRF01 (3/119, 2.5%), CRF01//B (1/119, 0.8%), B//CRF01 (1/119, 0.8%), CRF01/C//C (2/119, 1.7%), CRF01/CRF07//CRF07 (1/119, 0.8%), CRF01/C//CRF01 (1/119, 0.8%) and A1/G//H (1/119, 0.8%) (Table 2 and Table S2). The distribution of genotypes for the samples having only *gag* sequences were as follows: CRF01_AE (10/17, 58.8%), subtype B (2/17, 11.8%), CRF07_BC (2/17, 11.8%), CRF01/B’/C (1/17, 5.9%) and the two with only *env* sequences were both subtype B (2/17, 11.8%) (Table S2). Six *gag* sequences were located outside of the subtype/CRF clusters, suggesting that they might be intersubtype recombinants. Bootscanning analyses of the 6 *gag* sequences showed the following recombinant structures: 3 CRF01_AE/C, 1 CRF01_AE/B’/C, 1 CRF01_AE/CRF07_BC and 1 A1/G (Fig.S1). The demographic and genotypic characteristics of the 136 participants with available nucleotide sequences are summarized in Table 1.

**Table 1:** Demograpic and epidemiologic information of the study subjects and the distribution of HIV-1 genotypes.

					HIV-1 Genotypes^d^									
Characteristics	Total	B (n = 5)	B’ (n = 18)	C (n = 1)	CRF01_AE (n = 79)					CRF07_BC (n = 18)			CRF02_AG (n = 4)	URFs (n = 11)
					CRF01-1	CRF01-4	CRF01-5	JL.DG^e^	CRF01-NG^f^	CRF07-1	CRF07-2	CRF07-NG^g^		
**Sex** a														
M	111	5	6	1	0	20	53	1	0	5	8	2	1	9
F	25	0	12	0	1	2	1	0	1	1	0	2	3	2
**Ethnicity**														
Han	120	5	16	1	1	21	52	1	0	5	6	3	0	9
Korean	7	0	1	0	0	0	1	0	0	1	0	0	4	0
Manchu	3	0	0	0	0	1	0	0	0	0	2	0	0	0
Others^b^	5	0	1	0	0	0	1	0	1	0	0	1	0	1
Unknown	1	0	0	0	0	0	0	0	0	0	0	0	0	1
**Site of sampling**														
Changchun	92	3	5	0	1	21	45	1	0	4	7	0	1	4
Jilin	19	0	8	0	0	1	3	0	0	0	0	3	0	4
Yanbian	11	0	3	0	0	0	1	0	1	1	0	0	3	2
Tonghua	3	0	0	0	0	0	2	0	0	0	0	0	0	1
Baishan	4	0	2	0	0	0	2	0	0	0	0	0	0	0
Baicheng	3	1	0	0	0	0	0	0	0	1	0	1	0	0
Liaoyuan	2	1	0	1	0	0	0	0	0	0	0	0	0	0
Songyuan	2	0	0	0	0	0	1	0	0	0	1	0	0	0
**Risk group** c														
MSM	83	5	0	0	0	18	46	1	0	3	8	0	0	2
Hetero	43	0	13	0	1	4	8	0	1	3	0	3	3	7
FPD	5	0	4	0	0	0	0	0	0	0	0	0	0	1
IDU	3	0	0	1	0	0	0	0	0	0	0	1	1	0
MTCT	1	0	1	0	0	0	0	0	0	0	0	0	0	0
Unknown	1	0	0	0	0	0	0	0	0	0	0	0	0	1
**Total**	136	5	18	1	1	22	54	1	1	6	8	4	4	11

a Sex: M, male; F, female.

b Other: other ethnic minority including Yi, Dai, Lisu and Mongol.

c Risk Groups: MSM, men who have sex with men; Hetero, heterosexual; FPD, former plasma donor; IDU, injecting drug user; MTCT, mother-to-child transmission.

d HIV-1 Genotypes: genotypes were determined based on all sequences available.

e JL.DG: samples with different genotypic identification assigned to the *gag* P17–P24 and *env* C2–C4 regions were designated as discordant genotype and were abbreviated as JL.DG.

f CRF01-NG: ungrouped CRF01_AE lineage.

g CRF07-NG: ungrouped CRF07_BC lineage.

**Table 2:** HIV-1 genotype information of 12 subjects of Jilin province with new recombinants.

Sequence ID	Site ofsampling	Year ofsampling	Sex^a^	Age	Riskgroup	*p17*–*p24*	*C2–C4*	Genotype^b^
						(HXB2: 781-1836 nt)	(HXB2: 7002-7541 nt)	
JL.RF01	Tonghua	2010	M	38	Hetero	CRF01/B’/C	NA^d^	CRF01/B’/C
JL.RF02	Changchun	2010	M	23	MSM	CRF01-4	B	CRF01-4//B
JL.RF03	Changchun	2010	M	51	Hetero	CRF01-5/CRF07-2	CRF07-2	CRF01-5/CRF07-2
JL.RF04	cYKAP	2010	M	27	Hetero	CRF01/C	CRF01	CRF01/C//CRF01
JL.RF05	Jilin	2010	M	34	Hetero	CRF01/C	C	CRF01/C//C
JL.RF06	Jilin	2010	F	23	Hetero	CRF01/C	C	CRF01/C//C
JL.RF07	Jilin	2010	M	49	MSM	B	CRF01-5	B/CRF01-5
JL.RF08	Changchun	2011	M	68	Hetero	CRF07-2	CRF01-4	CRF07-2//CRF01-4
JL.RF09	Changchun	2011	M	21	Hetero	CRF07-2	CRF01-4	CRF07-2/CRF01-4
JL.RF10	Jilin	2011	F	41	FPD	A1/G	H	A1/G//H
JL.RF11	cYKAP	2011	M	unknown	unknown	CRF07-1	CRF01-5	CRF07-1//CRF01-5
JL.DG01	Changchun	2011	M	24	MSM	CRF01-5	CRF01-4	CRF01-5//CRF01-4

a Sex: M, male; F, female.

b Genotype was determined based on both *gag* and *env* gene region sequences.

c YKAP: Yanbian Korean Autonomous Prefecture.

d NA: not available.

Our previous national-wide study [21] identified 7 major, distinct lineages among China’s CRF01_AE epidemic. We found that some Jilin province CRF01_AE strains sub-clustered with CRF01-4 (22/79, 27.8%) and CRF01-5 (54/79, 68.4%) lineages, predominantly found among MSM in both this and our previous study (Fig. 1A and Fig. 2A) [21], and just one strain of CRF01-1 (1/79, 1.3%), commonly found among heterosexuals and IDUs in the previous study, but in a heterosexual transmission in this study. In addition, one sequence did not group with other known transmission clusters (1/79, 1.3%) (designated as CRF01-NG). In the present study, we found 1 CRF01_AE recombinant consisting of CRF01-5 in *gag* and CRF01-4 regions in *env*.

We also identified two strongly supported monophyletic transmission sub-clusters of CRF07_BC among sexual transmissions based on both *gag* and *env* sequences; CRF07_BC-1 was previously identified [24]–[26]. However, in the current study, a new lineage, designated CRF07_BC-2, was identified only in MSM from Jilin and Songyuan. Among 18 CRF07_BC strains identified in the present study, 6 (33.3%) and 8 (44.4%) belonged to CRF07-1 and CRF07-2 respectively, and 4 (22.2%) CRF07_BC strains were located outside of these two clusters (Fig. 1B and Fig. 2B and Table 1).

### HIV-1 genotype distribution by risk group

As summarized in Table 1, a socio-demographic study was performed to better characterize the distribution of HIV-1 genotypes. We found intermixing of HIV-1 subtypes/CRFs in sexual transmissions (MSM and heterosexual) accounting for 92.6% of the genotyped infections. Based on HIV-1 *gag* P17–P24 and/or *env* C2–C4, among the MSM population, CRF01_AE, CRF07_BC, subtype B and recombinants accounted for 78.3%, 13.3%, 6.0% and 2.4% of HIV-infections, respectively. The greatest diversity of HIV subtypes and recombinant viruses was observed among the heterosexual population with CRF01_AE, subtype B’, recombinants, CRF07_BC and CRF02_AG accounting for 32.6%, 30.2%, 16.3%, 14.0% and 7.0% of the infections, respectively. Recombinants were primarily found among the heterosexual transmissions compared to other risk groups, accounting for 63.6% (7/11) of all recombinants (Table 1 and Table 2). Subtype B’ (13/18, 72.2%) and CRF02_AG (3/4, 75.0%) were most frequently identified among the heterosexual population; none were found in our 83 MSM. CRF01_AE was the primary HIV-1 genotype found among the MSM and heterosexual populations, while no CRF01_AE strains were identified in our 3 IDUs. Three different genotypes were detected among our 3 IDUs: subtype C (33.3%), CRF02_AG (33.3%) and CRF07_BC (33.3%).

### HIV-1 genotype distribution by geographic region

As shown in Table 1 and Figure 2, most HIV sequences were collected in Changchun (92/136, 67.6%), Jilin (19/136, 14.0%) and Yanbian Korean Autonomous Prefecture (11/136, 8.1%). Six out of the 7 identified subtypes/CRFs of HIV-1 were found in Changchun. Most of these HIV-infections were found in MSM (72/92, 78.3%) and heterosexuals (19/92, 20.7%), and the two predominant genotypes were CRF01_AE (68/92, 73.9%) and CRF07_BC (11/92, 12.0%). Seven out of 8 (87.5%) of the newly identified CRF07-2 strains were identified in Changchun, and the other CRF07-2 infection was found in the neighboring city of Songyuan. Of note, 95.5% of CRF01-4 and 83.3% of CRF01-5 co-circulated in Changchun, therefore, it was not surprising that we discovered a recombinant genotype (designated JL.DG01) comprised of gene regions from both CRF01-4 and CRF01-5. However, it should be noted that 68% of all our sequences were from Changchun.

**Figure 3:**
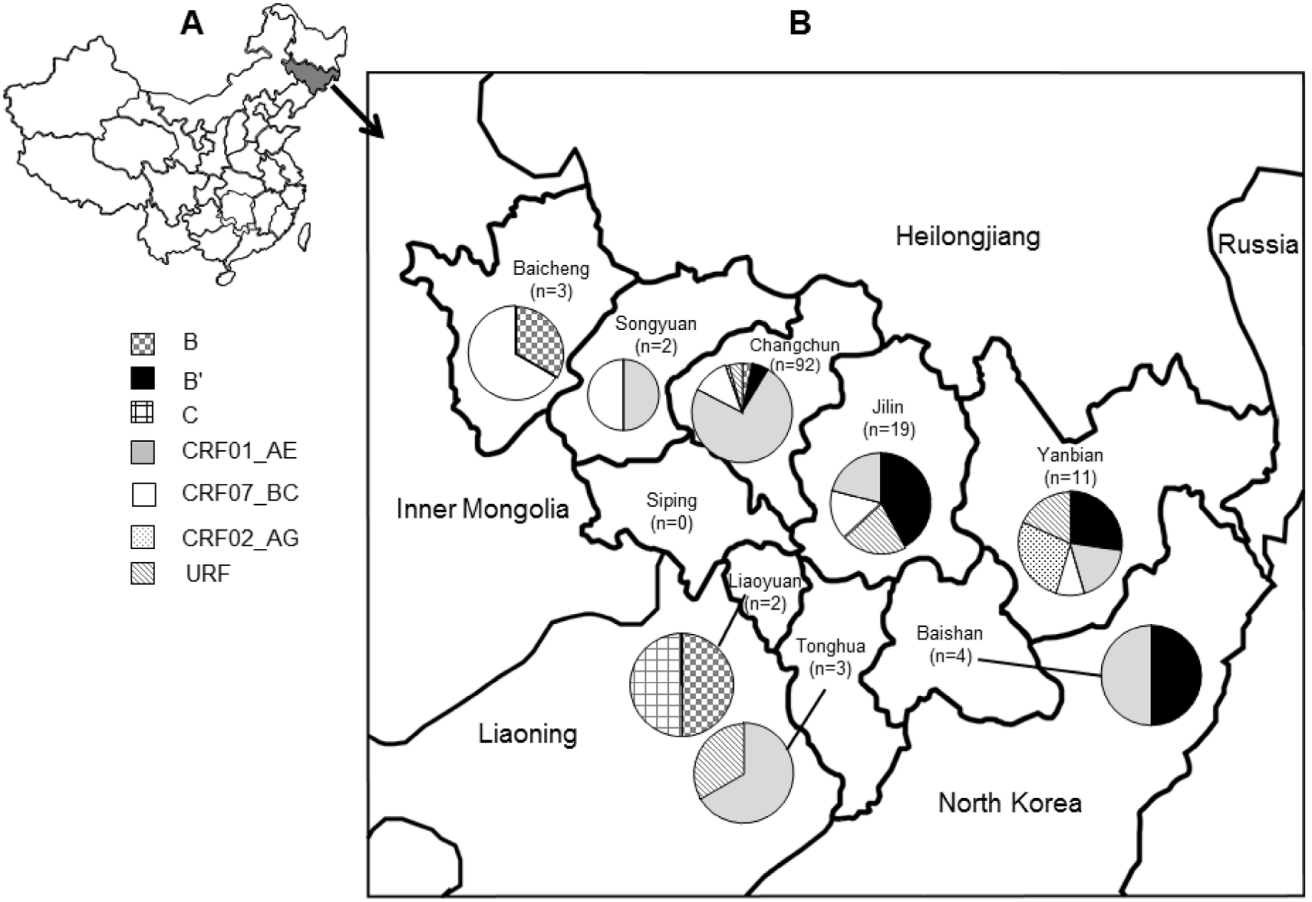
Maps of the study regions and HIV-1 genotype distribution of each geographical region in Jilin province of northeastern China. At the top-left corner of the figure is map of China (A), Jilin province of northeastern China is marked in gray. The map on the right of the figure represents Jilin province of northeastern China (B), the geographical location of the nine regions within Jilin province and the numbers of samples with genotypes from each region are shown. Pie charts show the distribution of HIV-1 genotypes in each region of Jilin province of northeastern China. The symbols representing different HIV-1 genotypes are shown at the left of the figure.

In the city of Jilin, heterosexual (9/19, 47.4%) and FPD (5/19, 26.3%) were the two major risk groups for HIV-infections with subtype B’ (8/19, 42.1%), followed by CRF01_AE (4/19, 21.1%), URFs (4/19, 21.1%) and CRF07_BC (3/19, 15.8%). In Yanbian Korean Autonomous Prefecture, the major risk group was heterosexual transmission (10/11, 90.9%), which was comprised of subtype B’ (3/11, 27.3%), CRF02_AG (3/11, 27.3%), CRF01_AE (2/11, 18.2%), recombinant viruses (2/11, 18.2%) and CRF07-1 (1/11, 9.1%). The sampling size in the remaining cities was very small (Table 1, Fig. 3).

## Discussion

Here we describe the most comprehensive HIV-1 molecular epidemiologic investigation, to date, on the characteristics and trends of the HIV/AIDS epidemic in the Jilin province of northeastern China between 2010 and 2011. Jilin province, as well as most of China, is experiencing an increasingly complex HIV epidemic. We identified subtypes B, B’, and C, CRF01_AE (including lineages CRF01-1, CRF01-4 and CRF01-5), CRF02_AG, CRF07_BC (CRF07-1 and the newly identified lineage CRF07-2), and recombinant viruses; CRF01_AE was the predominant genotype in both heterosexual and MSM sequences.

In a previous nation-wide study [21], we identified 7 unique, strongly supported, phylogenetic sub-clusters or lineages among the CRF01_AE radiation nation-wide. These lineages were partially segregated by geographic regions of China and different risk groups. The first three lineages (CRF01-1through CRF01-3) were prevalent among IDUs and heterosexuals from the south and southwest, lineages CRF01-4 and CRF01-5 were primarily found in MSM from northern cities, and sub-clusters CRF01-6 and CRF01-7 were found in heterosexuals from southern China. In the current study, we identified three of these CRF01_AE lineages. CRF01-4 and CRF01-5 were found among sexual transmissions, both heterosexual and MSM, while the one CRF01-1 virus was identified in a heterosexual male.

Previous studies have found that there was a large and strongly supported transmission sub-cluster of CRF07_BC strains among MSM in Beijing, Liaoning, and Shijiazhuang within the CRF07 radiation [22]–[24], we designated this CRF07_BC sub-cluster as CRF07-1. In this current study, we found the route of transmissions for CRF07-1 had expanded to include heterosexuals as well as MSM in Jilin province. Furthermore, a new statistically supported, monophyletic transmission cluster of CRF07_BC (designated CRF07-2) was identified among seven MSM from Changchun and one MSM from Songyuan, which is geographically adjacent to the northwestern portion of Changchun. Although the sample sizes are still fairly small relative to the epidemic, CRF07-1 has spread throughout much of Jilin province and is transmitted by both heterosexuals and MSM, while CRF07-2 has only been found among MSM in Changchun and neighboring Songyuan.

Originally subtype B’ infections in China were identified primarily among IDU in Yunnan province and subsequently among FPD and heterosexual in inland China, due to unhygienic commercial plasma collections during the early to mid-1990s, after which the practice was banned [25]. The majority of subtype B’ infections are now found in the heterosexual population [2]. This shift in risk groups is most likely the result of national policies in China that strictly regulated blood donations, which began in the late 1990s [26] and the fact that the HIV-infected FPD were spreading subtype B’ viruses through heterosexual transmissions to their sex partners. Indeed, we found no subtype B’ infections among our 83 MSM from Jilin province. While these comparisons of genotypes and risk groups are interesting, and appear to indicate certain trends, it must be highlighted that the samples were collected by convenience and do not represent a random sampling. An unknown degree of sampling bias is likely.

In the present study, we also detected a new statistically supported monophyletic cluster of 4 CRF02_AG uniquely associated with Korean ethnicity and heterosexuals near the Jilin-North Korean border (Yanbian Korean Autonomous Prefecture). The exception was an IDU in Changchun, who was also a citizen of Yanbian Korean Autonomous Prefecture but was imprisoned in Changchun. In a previous study, it was also showed that CRF02_AG predominated among heterosexuals in Cina, accounting for at least 68.8% of all detected CRF02_AG in a nationwide cross-sectional study of HIV-1 epidemic in China. In light of the fact that the four study participants were all of Korean ethnicity, it is tempting to speculate that the CRF02_AG cluster detected in the present study originated in Yanbian Korean Autonomous Prefecture. It is the first report of CRF02_AG forming a transmission cluster in a local resident population in China. In the past, CRF02_AG had only been found in migrating workers from West Africa [27], [28].

Among the complex mix of subtypes and CRFs that co-circulate in China, it was not surprising to find the emergence of *gag* and *gag/env* recombinants. All but 1 (90.9%) of the 11 recombinants we found contained at least a portion of CRF01_AE, which is consistent with CRF01_AE being the predominant genotype among both heterosexual and MSM transmissions. Overall, the highest HIV-1 genotype diversity was observed among the heterosexual population, however, we only had three IDU samples. If we had a larger sampling of IDUs, we may have found a larger genotype diversity in this risk group too, due to the presence of both needle sharing and sexual routes of infection. In fact, from the three Jilin IDU sequences, we found three different genotypes, subtype C, CRF07_BC, and CRF02_AG, supporting the hypothesis that if more samples were available from IDUs, we might have found a genotype diversity at lease as high as that seen in heterosexual transmissions.

China’s HIV epidemic is very heterogeneous, composed of a series of overlapping local sub-epidemics defined by risk groups, as well as temporal and/or spatial variation [29]–[34]. Detecting these sub-epidemics can be challenging because HIV diagnosis may take place many years after infection, and because of reliance on risk group self-reporting. IDU, MSM, and heterosexual risk factors are complicated by frequent ‘bridging’ between risk groups, caused by concurrent risk behaviours. IDUs constitute a high-risk group, which can facilitate HIV spread to lower risk populations through sexual risk factors [35]–[43]. While MSM has become the predominant route of HIV transmission [40], [44],[45], it is common for MSM in China to be bisexual [41]–[43], because of traditional values and family expectations; 17–35% of MSM are married [46]–[49] and more than 70% will be married in their lifetime [48], [50]. Furthermore, an estimated 8.3% of MSM used drugs in the past 6 months and 5–25% sold sex to other men [50]–[56].

In conclusion, our study indicates that the HIV epidemic in Jilin province is very complex; effective control will require an understanding of the dynamics that drives the spread of HIV through social/sexual transmission networks [57], [58]. Future molecular epidemiologic studies need to focus on collecting detailed behavioral and socio-demographic information that will allow the characterization of common behavioral risk factors for each of the identified social/sexual transmission clusters. Since transmission clusters significantly drive the HIV-1 epidemic in China, characterizing the specific, common risk behaviors for these networks will help target intervention strategies.

## Supporting Information

Figure S1 **Bootscanning analyses of 6** ***gag*** **sequences of HIV-1 URFs strains isolated from Jilin province of northeastern China.** The conditions used for bootscanning analyses are described in Methods. The representative subtypes/CRFs reference sequences with corresponding colors are shown at the bottom right of the figure.
(TIF)

Table S1 **Demographic and epidemiologic characterization of the study participants.**
(DOC)

Table S2 **Demographic and genotypic characterization of the study participants.**
(XLS)
