# Supplementary figures and images for: Molecular Epidemiology of HIV-1 in Jilin Province, Northeastern China: Emergence of a New CRF07_BC Transmission Cluster and Intersubtype Recombinants
Source: PLoS One. 2014 Oct 30;9(10):e110738. doi: 10.1371/journal.pone.0110738 (PMC4214716; doi:10.1371/journal.pone.0110738)

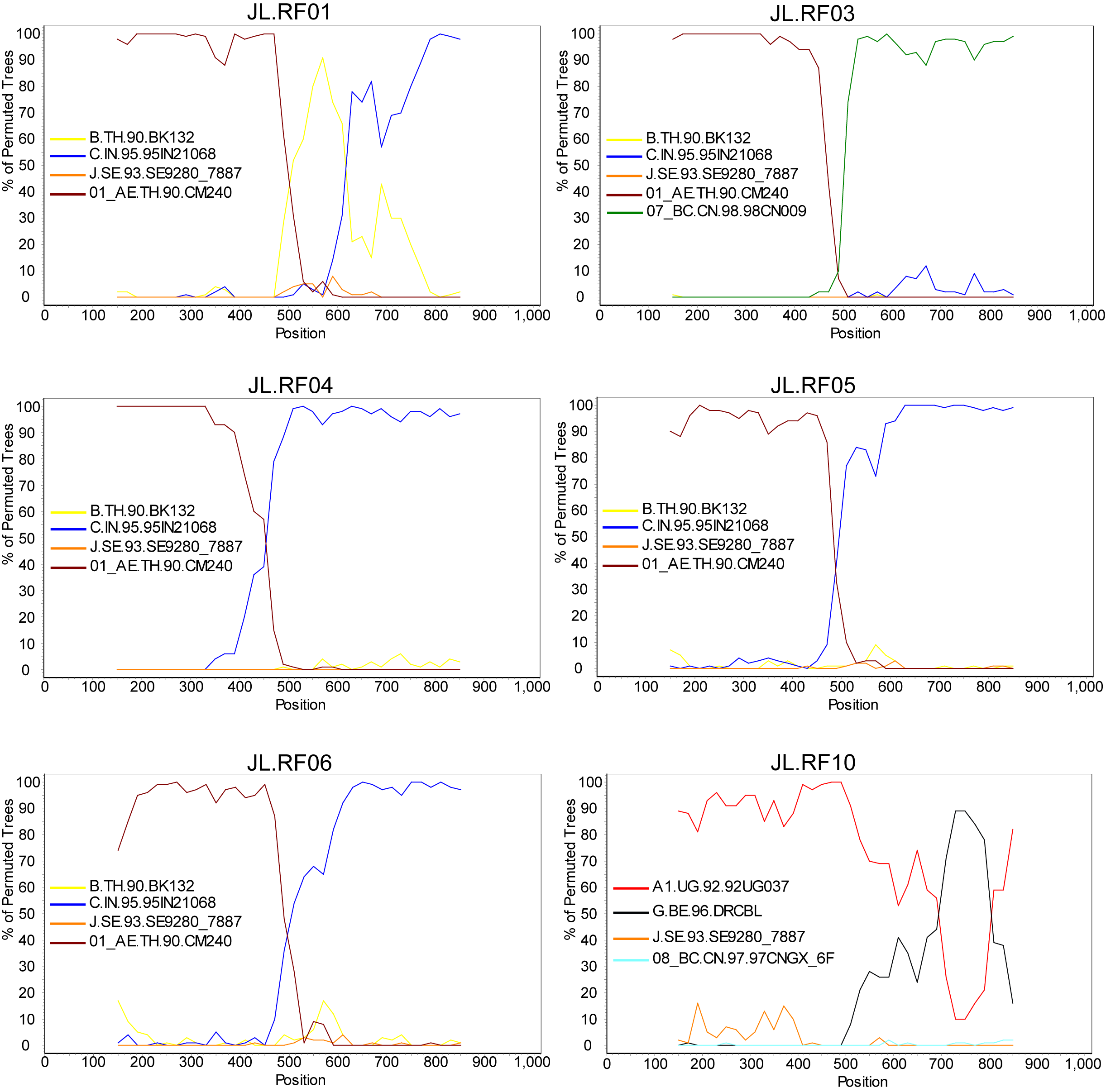

Supplement: Figure S1 — Bootscanning analyses of 6 gag sequences of HIV-1 URFs strains isolated from Jilin province of northeastern China. The conditions used for bootscanning analyses are described in Methods. The representative subtypes/CRFs reference sequences with corresponding colors are shown at the bottom right of the figure. (TIF) [file pone.0110738.s001.tif]
