# Supplementary material for: Molecular Epidemiology of HIV-1 in Jilin Province, Northeastern China: Emergence of a New CRF07_BC Transmission Cluster and Intersubtype Recombinants
Source: PLoS One. 2014 Oct 30;9(10):e110738. doi: 10.1371/journal.pone.0110738 (PMC4214716; doi:10.1371/journal.pone.0110738)
Supplement: Table S1 — Demographic and epidemiologic characterization of the study participants. (DOC) [file pone.0110738.s002.doc]

**Table S1. Demographic and epidemiologic information of the study participants.**

| **Characteristics** | **No. of Collected subjects from each demographic**  **N (%)** | **No. of genotyped subjects**  **from each demographic**^d^  **N (%)** |
| --- | --- | --- |
| **Total** | 189 (100.0) | 136 (100.0) |
| **Sex**^a^ |  |  |
| M | 154 (81.5) | 111 (81.6) |
| F | 35 (18.5) | 25 (18.4) |
| **Age** |  |  |
| <26 | 35 (18.5) | 31 (22.8) |
| 26-45 | 105 (55.6) | 73 (53.7) |
| 46-60 | 37 (19.6) | 24 (17.6) |
| >60 | 11 (5.8) | 7 (5.1) |
| Unknown | 1 (0.5) | 1 (0.7) |
| Mean ± SD^b^ | 37.3±12.9 | 35.7±13.0 |
| **Ethnicity** |  |  |
| Han | 167 (88.4) | 120 (88.2) |
| Korean | 11 (5.8) | 7 (5.1) |
| Manchu | 3 (1.6) | 3 (2.2) |
| Dai | 2 (1.0) | 1 (0.7) |
| Yi | 2 (1.0) | 2 (1.5) |
| Hui | 1 (0.5) | 0 |
| Lisu | 1 (0.5) | 1 (0.7) |
| Mongol | 1 (0.5) | 1 (0.7) |
| Unknown | 1 (0.5) | 1 (0.7) |
| **Marital Status** |  |  |
| Unmarried | 81 (42.9) | 70 (51.5) |
| Married | 63 (33.3) | 35 (25.7) |
| Divoiced/Widowed | 44 (23.3) | 30 (22.1) |
| Unknown | 1 (0.5) | 1 (0.7) |
| **Educational background** |  |  |
| Illiteracy | 6 (3.2) | 5 (3.7) |
| Primary/Middle school | 95 (50.3) | 68 (50.0) |
| High/Secondary school | 56 (29.6) | 35 (25.7) |
| Junior or above | 31 (16.4) | 27 (19.9) |
| Unknown | 1 (0.5) | 1 (0.7) |
| **Year of diagnosis** |  |  |
| 2008 | 76 (40.2) | 52 (38.2) |
| 2009 | 17 (9.0) | 12 (8.8) |
| 2010 | 96 (50.8) | 72 (52.9) |
| **Year of Sampling** |  |  |
| 2010 | 93 (49.2) | 64 (47.1) |
| 2011 | 96 (50.8) | 72 (52.9) |
| **Site of Sampling** |  |  |
| Changchun | 125 (66.1) | 92 (67.6) |
| Jilin | 20 (10.6) | 19 (14.0) |
| Yanbian | 15 (7.4) | 11 (7.4) |
| Tonghua | 10 (5.3) | 3 (2.2) |
| Liaoyuan | 4 (2.1) | 2 (1.5) |
| Baishan | 4 (2.1) | 4 (2.9) |
| Baicheng | 4 (2.1) | 3 (2.2) |
| Songyuan | 4 (2.1) | 2 (1.5) |
| Siping | 3 (1.6) | 0 |
| **CD4^+^ T Cell Count (cells/µl)** |  |  |
| >500 | 31 (16.4) | 23 (16.9) |
| 200-500 | 120 (63.5) | 90 (66.2) |
| <200 | 38 (20.1) | 23 (16.9) |
| Median | 380 | 369 |
| **Risk Group**^c^ |  |  |
| MSM | 107 (56.6) | 83 (61.0) |
| Hetero | 65 (34.4) | 43 (31.6) |
| FPD | 9 (4.8) | 5 (3.7) |
| IDU | 3 (1.6) | 3 (2.2) |
| BT | 2 (1.1) | 0 |
| MTCT | 1 (0.5) | 1 (0.7) |
| Unknown | 2 (1.1) | 1 (0.7) |

^a^Sex: M, male; F, female.

^b^SD: Standard deviation.

^c^Risk group: MSM, men who have sex with men; Hetero, heterosexual; FPD, former plasma donor; IDU, injecting drug user; BT, blood transfusion recipient; MTCT, mother-to-child transmission.

^d^No. of genotyped subjects from each demographic: the HIV-1 genotype of 136 samples was determined based on at least one fragment of *gag* P17-P24 and *env* C2-C4 sequences and the 136 samples were used in the following analyses.
